# Supplementary material for: Improved Method for Efficient Generation of Functional Neurons from Murine Neural Progenitor Cells
Source: Cells. 2021 Jul 26;10(8):1894. doi: 10.3390/cells10081894 (PMC8392300; doi:10.3390/cells10081894)
Supplement: Supplementary file 1 [file cells-10-01894-s001.zip › cells-1194841-supplementary.pdf]

cells

MDPI

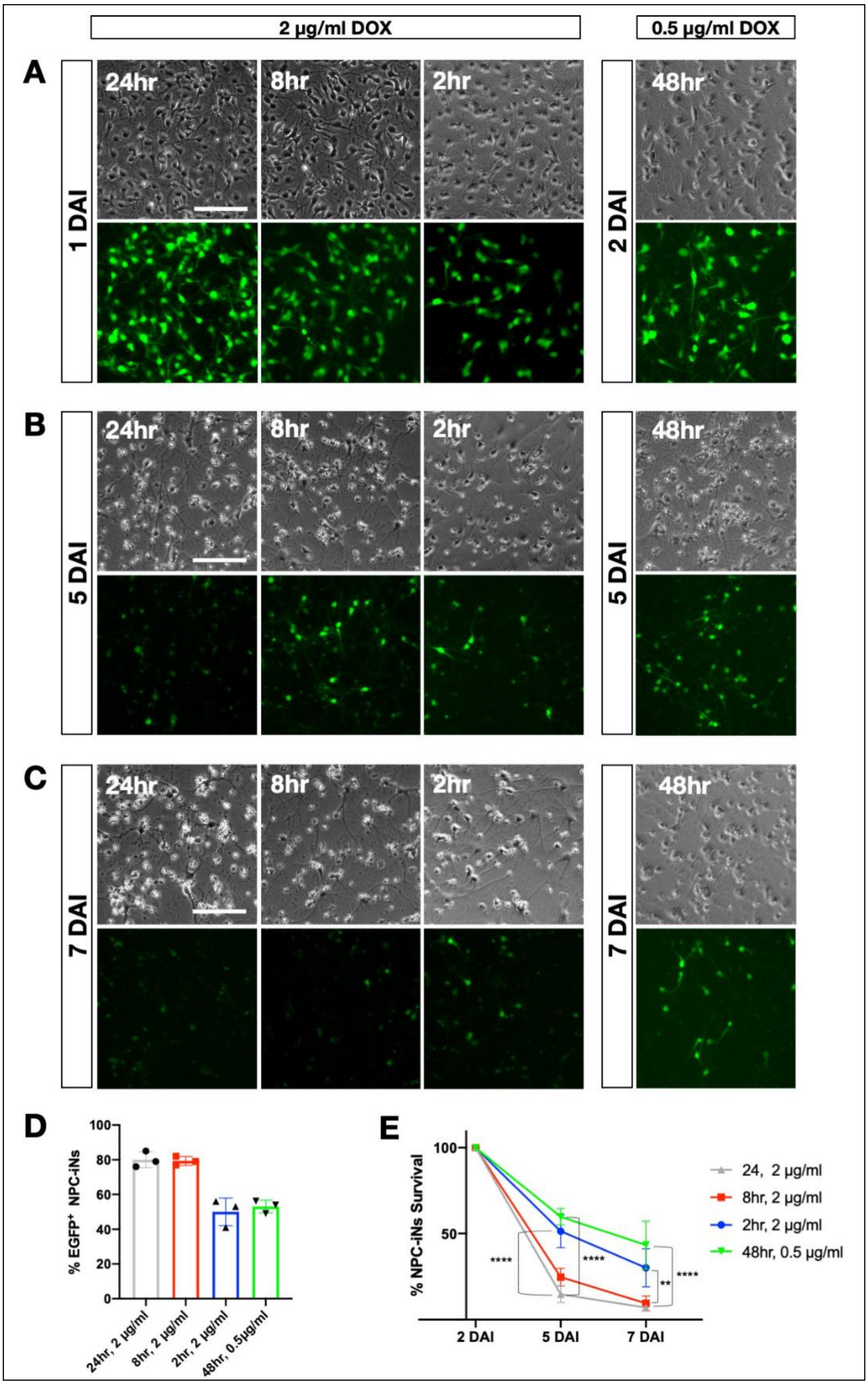

Supplemental Figure 1. Optimization of DOX-treatment. (**A**–**C**) Bright-field and EGFP images showing NPC-iNs (1/2, 5, and 7 DAI) induced with 2 µg/mL DOX for durations of 2 h, 8 h, 24 h or with 0.5 µg/mL DOX for 48 h. Scale bar = 100 µm. (**D**) Quantification of the fraction of EGFP⁺ NPC-iNs at 1 DAI (2 µg/mL DOX) or at 2 DAI (0.5 µg/mL DOX). (**E**) Quantification of the fraction of NPC-iNs surviving at 5 and 7 DAI. Two-way ANOVA was performed to test the effects of DOX timing (2 h, 8 h, 24 h, 48 h) and numbers of DAI (1 or 2/5/7 DAI) on the survival of induced neurons (effect of DOX timing, $p = 0.0004$; numbers of DAI, $p < 0.0001$), followed by Tukey's multiple comparison test to observe differences between groups (N = 4, **** $p < 0.0001$, ** $p < 0.001$). Data are presented as mean ± s.d.

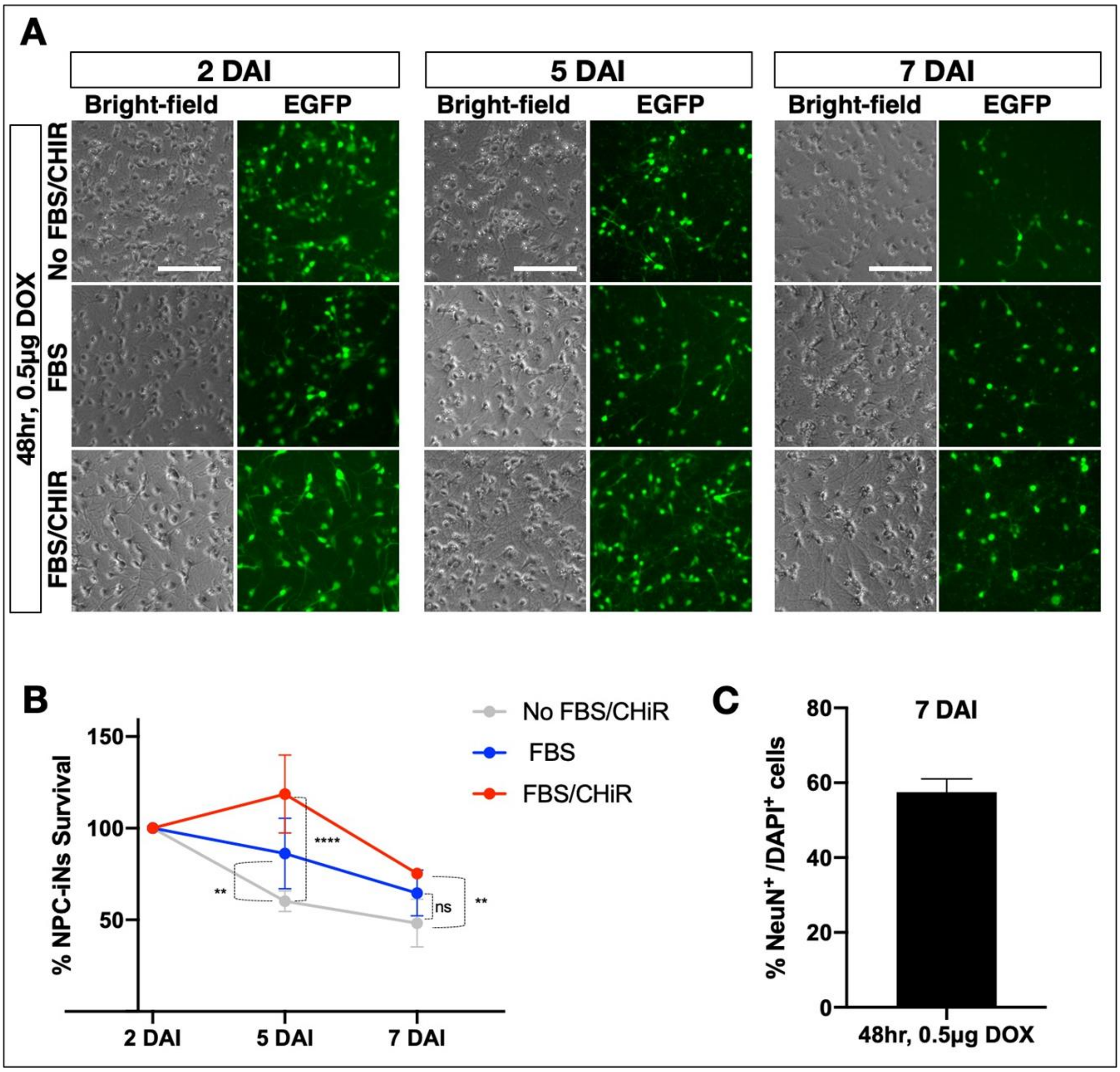

Supplemental Figure 2. Improvement of survival rate of NPC-iNs. (**A**) Bright-field and EGFP images showing NPC-iNs (2, 5, and 7 DAI) induced with 0.5 µg/mL DOX for 48 h and treated with no treatment, only 0.1 % FBS or both 0.1% FBS and CHIR. Scale bars = 100 µm. (**B**) Quantification of the fraction of NPC-iNs surviving at 5 and 7 DAI after DOX induction and treatment with no FBS/CHIR, only FBS or FBS/CHIR. Two-way ANOVA was performed to compare the effect of different treatments (No FBS/CHIR, FBS, FBS/CHIR) and numbers of DAI (2/5/7 DAI) on the survival of iNPC-iNs. Significant effect of different treatments and numbers of DAI ($p < 0.0001$) on cell survival were observed. This was followed by Tukey's multiple comparison test to observe significant differences within groups (N = 3, ** $p < 0.005$, **** $p < 0.0001$, ns = not significant). (**C**) Quantification of the neuronal yield at 7 DAI for NPC-iNs induced with 0.5 µg/mL DOX for 48 h based on the fraction of NeuN-positive cells among DAPI-positive cells (504 cells from three independent experiments, 57.5 ± 3.6%). Data are presented as mean ± s.d. ns = not significant.

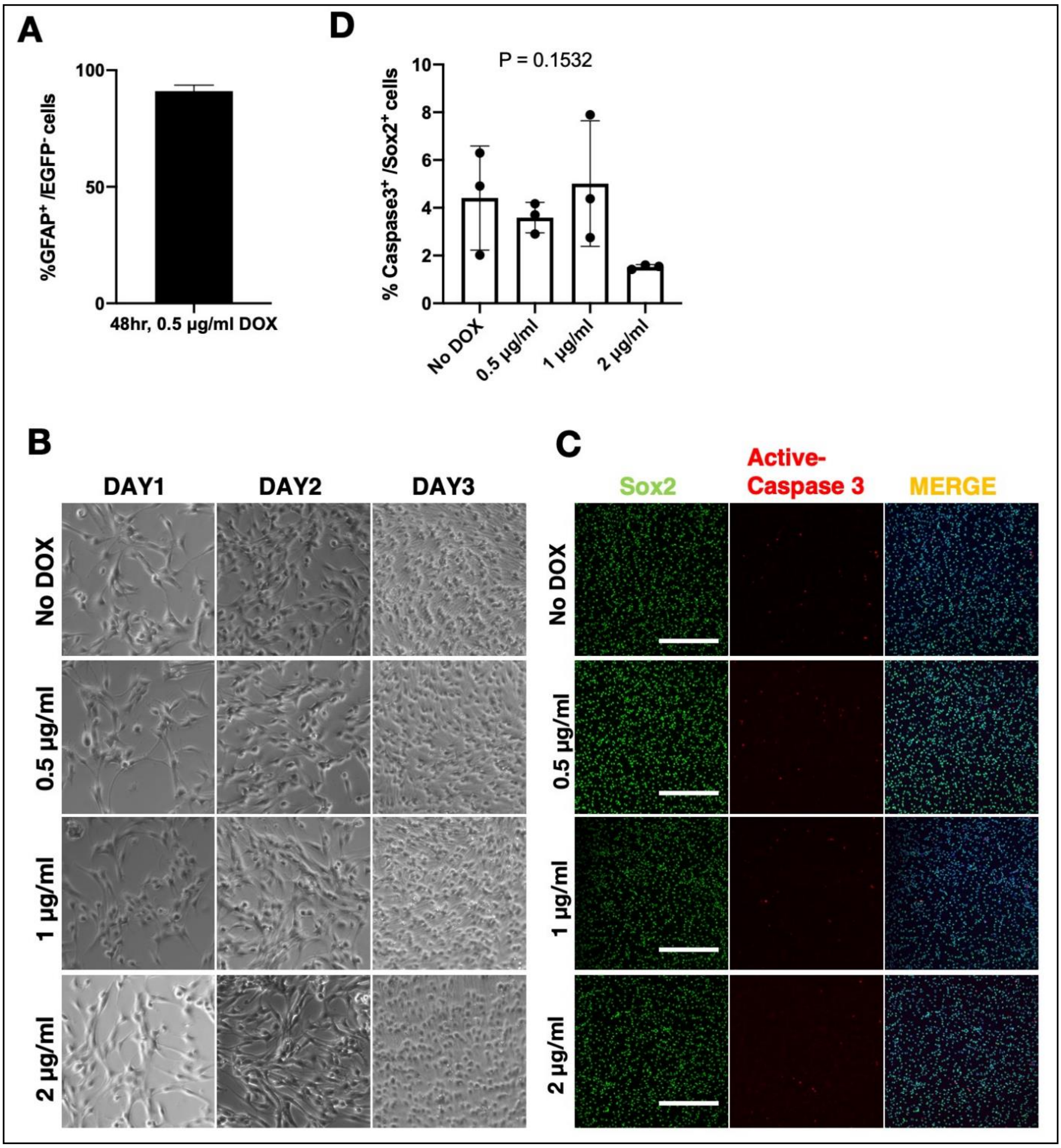

Supplemental Figure 3. Verification of experimental conditions. (**A**) Quantification of EGFP negative cells with a glial marker GFAP with 0.5 µg/mL DOX for 48hr (504 cells from three independent experiments, 91.1 ± 2.6%). (**B**) Effect of DOX on mNPC. Brightfield images of proliferating naive mNPCs treated with different concentrations of DOX. No obvious changes on the rate of proliferation were observed. Scale bars = 100 µm. (**C**) Images of naive mNPCs three-days post treatments with different concentrations of DOX and stained for mNPC marker Sox2 (green), cell death marker active-caspase 3 (red) and DAPI (blue). (**D**) Quantification of the fraction of active-caspase3 positive mNPCs after three-days treatment with either No DOX (4.4 ± 2.2%), 0.5 µg/mL DOX (3.6 ± 0.6%), 1 µg/mL DOX(5.0 ± 2.6%), or 2 µg/mL DOX (1.5 ± 0.1%). No significant difference in the fraction of active-caspase 3 positive cells (N = 3, $p = 0.1532$, one-way ANOVA). Data are presented as mean ± s.d.
